# Early lexical processing of Chinese words indexed by Visual Mismatch Negativity effects

**DOI:** 10.1038/s41598-018-19394-y

**Published:** 2018-01-22

**Authors:** Dawei Wei, Margaret Gillon Dowens, Taomei Guo

**Affiliations:** 1 School of Foreign Languages, Lanzhou Jiaotong University, Lanzhou, China; 2 Cognitive Neuroscience of Language Laboratory, University of Nottingham Ningbo China, Ningbo, China; 3 State Key Laboratory of Cognitive Neuroscience and Learning & IDG/McGovern Institute for Brain Research, Beijing Normal University, Beijing, China

## Abstract

Although Mismatch Negativity (MMN) effects indicating early, automatic lexical processing have been reported in the auditory language modality, so far these have not been reliably obtained in MMN studies of visual word recognition. The present study explores this discrepancy by investigating whether visual MMN (vMMN) effects can be obtained in written Chinese single-character word recognition. While participants were engaged in a non-linguistic distraction task, we measured Event-Related Potentials (ERPs) time-locked to perifoveally-presented real and pseudo- characters matched in overall visual-orthographic attributes. VMMN was defined as significant difference between the ERPs to characters presented as deviants or as standards in a context of non-characters. For the native Chinese readers, af ter sub-lexical structural detection from 120–160 ms, only real characters elicited vMMN at the interval of 170–210 ms, suggesting that lexical information in Chinese words is processed early and automatically. In a later window of 340–380 ms, both real and pseudo- characters yielded vMMNs. In a control group of non-Chinese participants, no evidence of vMMN was found for either real or pseudo-characters. Taken together, these results suggest that long-term memory representations for real characters may enable their early processing even in unattended conditions.

## Introduction

In our modern societies, we are constantly exposed to written language presented through different media such as posters and street signs, mobile phones and the internet, as well as magazines and books. Reading is, in fact, a remarkable human achievement but in skilled readers it is so effortless and reflex-like that normally we take it for granted, suggesting that reading comprehension in skilled readers occurs immediately and automatically, that is, without conscious attention or control. Basic to efficient reading is expertise in word recognition, which involves discriminating between word forms with various orthographic regularities, such as real existing words (e.g., work), and word-like forms which can share some orthography with real words but in fact do not exist (pseudowords or nonwords, e.g., wrok, krwo). In the psychology of language, there is considerable debate about exactly when and how such words are processed in the brain. Some researchers have claimed that lexical processing is late and controlled^1,2^. Other studies, however, seem to suggest that it is early and to some extent automatic^3,4^. So far, no definitive answer to this question has yet been provided. Nevertheless, examining this issue is critical to modelling word recognition^5,6^ and unveiling the nature of linguistic processing^7^. The present study therefore aims to contribute to this debate by investigating the early time-course and automaticity of word processing in Chinese. To examine this issue, we used the event-related brain potentials (ERPs) technique, which, with its millisecond-level time resolution, is a very useful tool to investigate fast-acting linguistic processes.

Two well-established ERP components, the N170 and the mismatch negativity (MMN) are of particular interest in this case, given their documented relevance to early lexical processing^8,9^. The N170 has been described as sensitive to the processing of orthographic patterns^8^. This negative waveform has a peak latency between 140 and 200 ms and is generally present at occipital-temporal/parietal regions. A number of ERP studies have associated the N170 with abstraction of orthographic information, rather than simple detection of the elementary visual features of letters or letter strings^8,10–15^. Across this large body of studies, overall a general pattern has emerged that in normal adult readers, words and word-like stimuli elicit larger N170 than do symbols, indicative of the N170 as a potential neural marker for reading expertise^16^. However, among the many unanswered questions about the nature of lexical processing is the issue of to what extent the N170 does indeed reflect lexical processing. In lexical decision tasks, both high-frequency^10^ and low-frequency^17^ words are reported to have larger N170 than pseudowords. In contrast, earlier studies did not report such effects of larger N170 in words than pseudowords^11^. The variety of tasks (perceptual detection, lexical decision, and semantic categorisation) used in these experiments arguably induce dif ferent task-related coping strategies and might therefore confound lexical processing per se. For example, while global attention to a letter string may be demanded in a font size judgment task^8^, a more local focus may be needed in a letter feature (lower case letter/thick letter) detection task^13^. This dif ference in task strategy may have contributed to effect differences in the two studies. Thus, it remains unclear to what extent information about lexicality is processed so early and whether this is affected by task demands. Another important issue is that usually, in previous word-related N170 studies, linguistic stimuli have been presented in the center of the screen, which in fact makes it very difficult not to attend them^18^ and the tasks themselves, for example, lexical decision, or semantic categorisation, have involved directly accessing different levels of linguistic analysis. In these conditions, it is difficult to provide definitive answers to questions about the earliness and automaticity of lexical processing^19^.

As well as the N170, in recent years another ERP component, MMN, has been widely used to tap into early-stage lexical processing. MMN is a negative ERP response elicited in oddball experiments by infrequent/deviant acoustic stimuli presented among frequent/standard sounds^20^. It is extracted by subtracting the ERPs to standard stimuli from the ERPs to deviant stimuli. MMN is usually observed at frontocentral electrodes, peaking about 100–250 ms af ter the onset of acoustic change, regardless of whether subjects attend to or ignore the stimuli^21^. Therefore, studies using a MMN design have the advantage of exploring cognitive processes independently of attentional interference and of task-related strategies^9^. Recent research has revealed that not only elementary auditory features such as sine wave tones, frequency and intensity, but also complex abstract features can result in MMN effects^22^. In the linguistic domain, it has emerged from a number of studies of auditory language processing that MMN is sensitive to lexical processing^4^. Specifically, acoustically presented real words have been shown to elicit enhanced MMN compared to acoustically and psycholinguistically-matched pseudowords, in several different languages^23–25^. The lexical enhanced auditory MMN, of ten seen as peaking as early as 100–200 ms, has been interpreted as reflecting pre-attentive activation of existing cortical memory representations for real words. These representations are conceptualised as distributed and robustly interconnected neuron populations^4^. The enhancement effect has thus been explained as a neural index of the long-term cortical memory traces for real words enabling activation of those words even in the absence of attentional resources^26^. In accordance with this theoretical account, lexical items, if supported by the corresponding cortical memory traces, should be activated automatically, regardless of the way the words are presented, whether auditorily or visually^27^.

Previous non-linguistic studies have in fact shown that there is an analogue of the auditory MMN in the visual modality, i.e., vMMN. vMMN is typically elicited by infrequent visual features such as color^28,29^, orientation^30^, and motion^31^. This component is usually observed in an interval around 130–400 ms at temporo-occipital electrodes. It has been generally accepted to be an objective indicator of early and automatic change detection in response to visual deviants^32,33^. Recently, however, there have also been some attempts to explore vMMN effects in language experiments. For example, Wang and colleagues investigated the extraction of phonology in Chinese character reading^34,35^. Chinese is a tonal language, with four meaning-bearing lexical tones embedded in character pronunciation. Homophone characters differing in orthography and semantics were visually presented in an oddball paradigm to participants who were asked to carry out a colour detection task^34^. Comparing deviants and standards of different lexical tones, vMMN was found at two intervals of 140–200 ms and 230–360 ms. It was reported by these authors that vMMN was elicited by a violation of the lexical tone pattern in the homophones, indicating that information about lexical tone was automatically extracted.

In another study, early and automatic lexical processing in Russian was investigated using a vMMN paradigm^27^. These authors briefly presented five oddball blocks of Russian words outside the attentional focus of participants. Each of the five blocks shared the same deviant-standard orthographic contrast. Participants were asked to carry out a primary distraction task which appeared in the middle of the screen. It was reported that real words produced a larger brain response than pseudowords as early as 110 ms af ter stimulus onset. Both real word and pseudo-word deviants were found to elicit vMMNs with similar amplitude, at 100–120 ms and 240–260 ms intervals. This seems to suggest that the vMMN was not sensitive to lexicality change. This is not consistent, however, with the above-mentioned auditory MMN studies in which real words are typically found to elicit enhanced MMN effects relative to pseudowords. Clearly, more studies are needed to clarify this issue.

In traditional MMN studies, the MMN component was normally derived by subtracting the ERPs to standards from those to deviants in the same block^32^. However, this practice may in fact confound MMN effects, due to the physical differences between deviants and standards. To solve this problem, later research has included an additional control condition where the role of deviant and standard stimuli in the traditional block is reversed. MMN is then computed by comparing identical stimuli acting as deviants and standards across different blocks. Known as the same-stimulus or identity MMN paradigm, the technique has been found to be a suitable way to control for physical difference and has been adopted in both auditory^36^ and visual^37,38^ MMN studies. The present study, therefore, will take advantage of this paradigm to explore vMMN effects in Chinese single-character word stimuli. In contrast to the linear arrangement of alphabetic letters in Indo-European languages, Mandarin Chinese characters, the fundamental building blocks of written Chinese, are composed of linguistic radicals which fit into a standard square-shaped block. The majority of characters in Chinese are compound characters, formed by linking two or more radicals together. In terms of overall graphical organisation, compound characters can be subdivided into three main types: lef t-right (e.g., ), top-bottom (e.g., ) and inside-outside (e.g., ). The horizontal lef t-right character is the dominant type^39^ and also the focus of the current study. Most horizontal characters consist of two parts, a semantic radical and a phonetic radical. As a general rule, the semantic and phonetic means metal, which is semantically radicals give rough cues respectively to the meaning and pronunciation of a character^39,40^. For example, the semantic radical  of the character  (/gang/, *steel*), means *metal*, which is semantically relevant to the character meaning, while its phonetic radical  is pronounced /gang/, carrying the same pronunciation as the host character.

Conventionally in lef t-right characters, a semantic radical is located on the lef t and a phonetic radical on the right, as seen in the above example. In other words, the predefined positional information has functional relevance to the character’s meaning and pronunciation. Still to take  for example, if the lef t-hand semantic radical  is placed on the right, the positional regularity is violated and the result, , is a non-character. In contrast, a pseudo-character has radicals which follow the positional rule, but which normally do not appear together. For example,  is a pseudo-character made up of the radicals  and  which are “correctly” placed respectively on the lef t and right side. It is a pseudo-character because although it is legally structured, there is no such compound character combining these two radicals in Chinese. By manipulating positional regularity and radical combination, a single set of phonetic and semantic radicals can be employed to build real, pseudo- and non- characters. Since normally radicals are fixed in size, these different types of characters occupy the same space and have identical visual complexity. Thus, Chinese orthographic patterns can allow us to exert control of physical variance between lexical stimuli of different types, which is important in a vMMN paradigm.

These unique features of Chinese orthography thus provide an opportunity to test for language MMN effects in the visual modality. The current study therefore aims to explore whether single-character words and word-like stimuli (real, meaningful characters and pseudo-characters) elicit vMMN and if so, to examine any differences between them. Previous auditory MMN studies have shown encoding of early, automatic lexicality information as indexed by enhanced MMN effects for real words compared to matched pseudowords^4^. Therefore, with native Chinese speakers, it is predicted that visually-presented lexicality information from Chinese single-character words may also be processed early and automatically, reflected in larger vMMN effects for real characters relative to pseudo-characters. A control group of non-Chinese readers was also tested. No vMMN effects are expected in this group, given that there will be no corresponding memory traces for Chinese radicals or characters.

## Results

### Behavioural result

The reaction times (ms) and accuracy rate (%) of judging the number of central red crosses were submitted to repeated measures ANOVAs of Group (native vs. non-native) by Block (four blocks). Results did not yield significant differences in reaction time and accuracy rate between blocks and between the two groups (ps > 0.05) (Table 1).

**Table 1:** Behavioural results (standard deviation in parenthesis).

	RN		NR		NP		PN	
	NT	NN	NT	NN	NT	NN	NT	NN
RT	442.6	474.3	458.2	482.3	445.5	475.0	449.7	473.3
	(65.2)	(59.4)	(74.1)	(55.2)	(54.0)	(46.6)	(58.8)	(46.5)
Acc. (%)	98.2	98.7	97.6	98.9	96.7	98.6	96.7	98.4
	(3.6)	(2.4)	(5.0)	(1.9)	(5.5)	(1.8)	(2.6)	(2.1)

*Note*. RN: standard real and deviant non- characters; NR: standard non- and deviant real characters; NP: standard non- and deviant pseudo- characters; PN: standard pseudo- and deviant non- characters. NT: native group; NN: non-native group. RT: reaction time (ms); Acc.: accuracy rate.

### ERP results

#### N170

The ANOVA of Group, Type, Lexicality and Hemisphere yielded a main effect of Group (F(1, 32) = 41.59, p < 0.001, partial η^2^ = 0.57, suggesting N170 was elicited only in the native but not in the non-native group (−1.98 vs. 1.35 µV). There was also a main effect of Hemisphere (F(1, 32) = 8.29, p = 0.007, partial η^2^ = 0.21), which was due to more negative N170 response in the lef t than in the right (−0.61 vs. −0.01 µV). There was no significant effects of Lexicality, or interactions with group and/or hemisphere did not reach significance (ps > 0.1), suggesting that real and pseudo- characters elicited similar N170 amplitude in the native group.

#### Comparing the ERPs to deviants and standards in the two groups

*120–160* *ms*: The ANOVA of Group, Type, Lexicality and Region revealed a main effect of Type (F (1, 32) = 6.19, p = 0.018, partial η^2^ = 0.16) and an interaction of Type, Group and Region (F(2, 64) = 6.26, p = 0.010, partial η^2^ = 0.16). Further analysis of the three-way interaction suggested differences between deviants and standards only for the native group, at Frontal (−1.12 vs. −0.52 µV, p = 0.002) and Central (−0.82 vs. −0.22 µV, p = 0.006) areas.

*170–210* *ms*: The ANOVA of Group, Type, Lexicality and Region showed a main effect of Type (F(1, 32) = 4.64, p = 0.039, partial η^2^ = 0.13) and interactions of Type and Group (F(1, 32) = 7.30, p = 0.011, partial η^2^ = 0.19), Type, Group and Lexicality (F(1, 32) = 4.87, p = 0.035, partial η^2^ = 0.13). Further analysis of the three-way interaction indicated that in the native group, differences between deviants and standards existed only for the real characters (−1.25 vs. 0.18 µV, p = 0.003), while there were no differences between deviant and standard stimuli either for real or pseudo- characters in the non-native group, ps > 0.1.

*280–320* *ms*: The ANOVA of Group, Type, Lexicality and Region suggested that no effects reached significance, ps > 0.05.

*340–380* *ms*: The ANOVA of Group, Type, Lexicality and Region suggested a main effect of Type (F(1, 32) = 9.00, p = 0.005, partial η^2^ = 0.22) and an interaction of Type and Group (F(1, 32) = 11.68, p = 0.002, partial η^2^ = 0.27). Further analysis of the interaction revealed differences between deviants and standards only in the native group (−0.74 vs. 0.30 µV, p < 0.001).

#### Comparing the vMMNs of real and pseudo- characters in the native group

*120–160* *ms*: The ANOVA of Lexicality and Region did not reveal a difference between real and pseudo- characters vMMN, p > 0.1.

*340–380* *ms*: The ANOVA of Lexicality and Region yielded a marginally significant effect of Lexicality (F(1, 16) = 3.64, p = 0.075, partial η^2^ = 0.19) suggesting that real characters vMMN tends to be larger than pseudo- characters’ (−1.52 vs. −0.58 µV).

## Discussion

The present study explored whether written Chinese lexical information is extracted automatically and rapidly, as indexed by vMMN effects. The ERPs to the same type of orthographic stimuli presented as deviants and standards across experimental blocks were compared in an identity paradigm. For the native group, first, real and pseudo- characters elicited an N170 component in the parietal-occipital area. Second, both types of characters elicited similar vMMNs in the windows of 120–160 ms. In 170–210 ms, only real characters elicited vMMNs while in 340–380 ms, both categories of characters yielded vMMN but real characters’ vMMN tended to be larger than that of pseudo-characters. In the control group of non-Chinese readers, there was no evidence of N170 and no evidence of vMMN. In the following, the results will be discussed in detail.

In the native group, within the first 200 ms af ter the linguistic stimulus onset, N170 of similar amplitude was elicited by both real and pseudo- characters over parietal-occipital areas. This result is in agreement with previous studies indicating that this component reflects expertise in written word processing in different writing systems^8,41,42^. Interestingly in the current study, this component was readily elicited even when participants’ attention was directed away from the visual stimuli located in the periphery. Similar results have also been found in recent vMMN studies of Chinese in attention-deprived contexts^34,35^ (but see another vMMN study of Russian words for an absence of the N170 effect^27^). Thus the presence of this component in our experiment seems to suggest that N170 is a robust neural marker of word literacy which can survive a strict non-attend design with a non-linguistic task. In the current study, both real and pseudo- character N170 were lateralised to the lef t hemisphere. This result is consistent with previous studies using different scripts such as Japanese Kanji and Kana words^42^, English words^43^ and Chinese characters^41^, confirming that N170 is lef t-lateralised in response to both alphabetic and logographic scripts. In the control group of non-native participants with very limited Chinese proficiency, no N170 was found in the current study. This result is in contrast to previous studies which reported N170 in learners of second languages (L2) regardless of their L2 proficiency levels^42,44^. This discrepancy may be mainly attributable to our task, which discouraged any attention to the peripheral characters.

In the initial stage (before 160 ms) of vMMN elicitation, both real and pseudo- characters yielded similar results in the native group. This may be attributed to the similar visual configuration of the two stimulus types. Real and pseudo- characters share radical identities and legal radical positions, in contrast to the non-characters, which have illegal positioning of radicals. This perceptual feature common to the two former categories is rapidly captured and is reflected neurophysiologically as vMMNs of similar amplitude. In order to examine whether the positional legality was decoded in this window, real and pseudo- characters ERPs were collapsed as legal characters in comparison with non-characters ERPs, for the native and non-native groups respectively. A mixed-design ANOVA of Group (native vs. non-native), Type (deviants vs. standards), position Legality (legal vs. non- characters) and Region (three regions as described in Methods) was carried out. This ANOVA yielded a four-way interaction (F(2, 64) = 5.16, p = 0.018, partial η^2^ = 0.14). Further analysis revealed that there were differences between deviants and standards for legal characters in the native group (Frontal, 0.09 vs. 0.56 µV, p = 0.057; Central, −0.85 vs. −0.21 µV, p = 0.007; Parietal, −1.11 vs. −0.53 µV, p = 0.003) while there were no differences between deviants and standards in the non-native group, ps > 0.1. This result indicates that the radical position legality was detected early only by the native group. The rapid processing of sub-lexical structure agrees well with previous studies in Chinese^45^ and in English^46^. For example, in an explicit character detection task, non-characters (“illegal pseudo-characters” in their study) were discriminated from legal pseudo-characters around 100 ms, as indexed by a larger P1 at lef t posterior electrodes^45^. Taken together, our data suggest that radical position is represented mentally and accessed at an initial stage of character recognition. Different from this Chinese study^45^, in the current experiment the early positional representation was detected even when participants’ attention was distracted away from the critical stimuli by a non-linguistic task. This automatic detection of such delicate spatial configurations of radicals can be attributed to the participants’ internalised knowledge of the character structure. However, knowledge of positional legality in Chinese characters, unlike elements of visual objects such as object shape and orientation, needs long-term language acquisition and explicit instruction. This type of explicit learning, or at least long-term exposure to the language, may be a prerequisite for mastery of Chinese radical spatial regularities and automatic processing of these. The absence of vMMN on the part of the non-native group lends support to this view, although they did show some knowledge of character structure at a behavioural level, as off-line they rated both real and pseudo characters significantly more real-character-like than non- characters.

As discussed above, early decoding of the positions of the radicals discriminates between real and pseudo- characters on the one hand, and illegal/non-characters on the other, as indicated by the vMMN effect before 160 ms. This early coarse orthographic analysis informs the following more fine-grained processing at the compound-character level. This is reflected in the presence of vMMN for real characters but an absence of vMMN for pseudo-characters in the interval of 170–210 ms. The locus of the real character vMMN advantage over pseudo-characters may lie in the integration of the radicals of the lexical stimuli. According to the part-based character encoding model^47^, in reading Chinese, the initial phase of stroke-based feature detection and radical processing feeds forward to the intermediate stage of integration of the lef t and right radicals, which distinguishes between real and pseudo- characters. In real characters, since the correct pairing of the two types of radicals can correspond with their established memory representations supported by robust underlying neural interconnections, successful activation of the lexical entries occurs. The visual input of the incorrectly-paired radicals in the pseudo-characters will, however, be lef t without further activation due to the lack of corresponding representations. It is noteworthy that in contrast to auditory lexical MMN studies, where pseudowords also elicit MMNs, albeit with a reduced amplitude^4,48^, no vMMN was found for the pseudo-characters in the interval of 170–210 ms. This difference may be related to the stimulus presentation modalities. In the auditory mode, with acoustic and phonological information unfolding over time, the competing representations of lexical neighbours of both real and pseudo- words may be activated before the real word is finally identified^49^. Thus, the partial activations for pseudowords may lead to a (reduced) MMN. In contrast, in a visual domain such as in the current study, real and pseudo- characters are presented as they are in entirety and immediately, leaving less opportunity to activate representations of relevant neighbours of the pseudo-characters, especially given the short stimulus duration in a non-attend design. As a result, activation of pseudo-characters may be minimal, and thus no reliable sign of vMMN appears. Recently, also in a visual modality, vMMNs of similar amplitude were found for Russian words and word-like stimuli^27^. This difference from our study could, however, be attributed to the different experimental configurations. The relatively small magnitude of stimulus change, as reflected in a delicate orthographic contrast (word-final consonant /K/ vs. /H/ in Russian), was shared among different lexical conditions. Too small a size of deviance change, especially in the context of the “rigorous distraction task” used of color circle tracking, can lead to weakened or no vMMN^28^. For the non-native readers in our study, no evidence of vMMNs was found for either real or pseudo- characters. This is likely due to their lack of sufficient Chinese language exposure and training. In fact, developing knowledge of the conventionalised radical pairings and of the thousands of different character forms requires extensive language experience and practice, as evidenced by the required years of rote memory and basic writing practice which are common in Chinese literacy education^50^.

**Figure 1:**
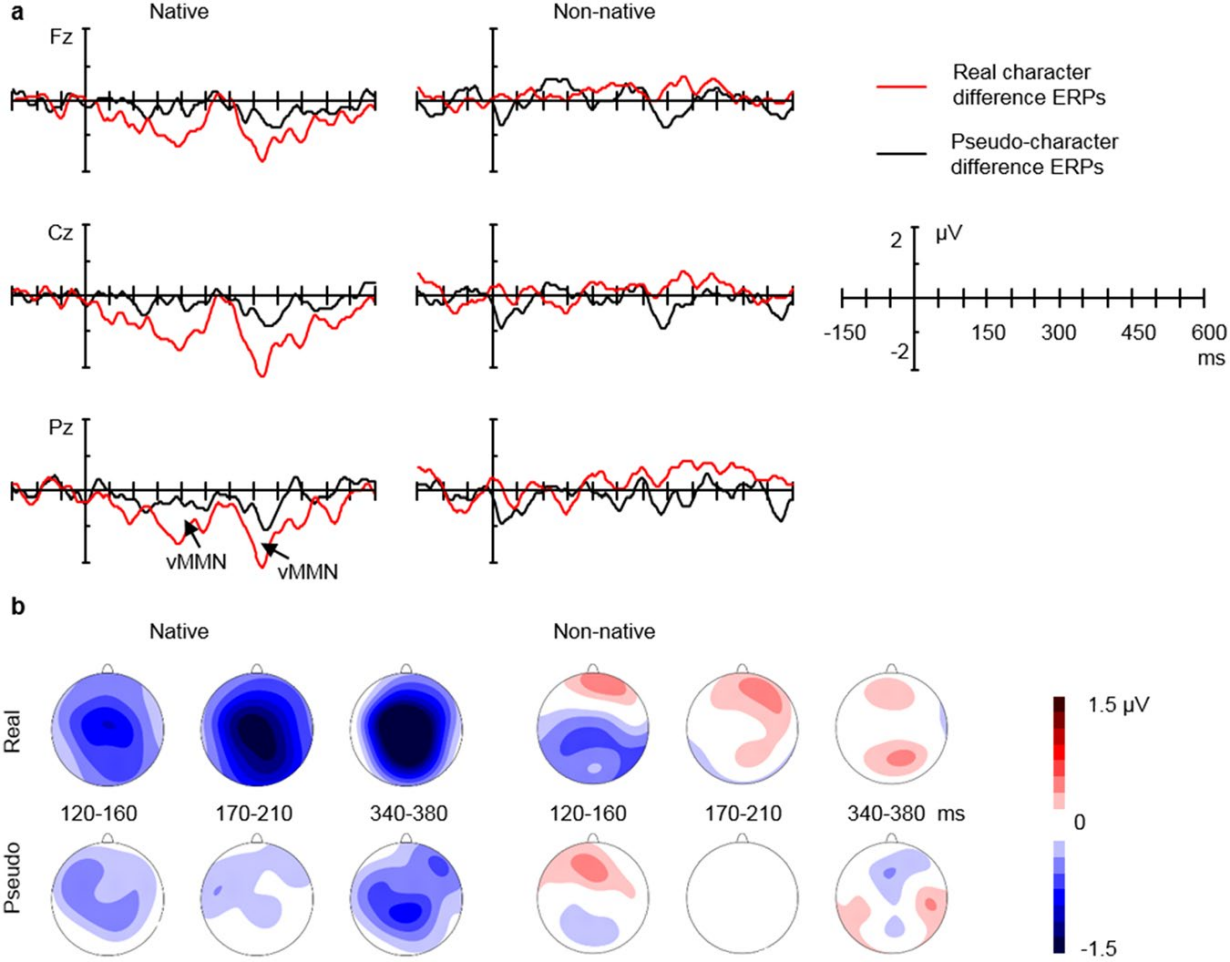
Comparison of real- and pseudo- character deviant-minus-standard difference ERPs. (**a**) Deviant-minus-standard difference waves of real characters (Red) and pseudo-characters (Black) at the representative electrodes Fz, Cz, Pz for the native (Lef t) and non-native (Right) group. Negativity is plotted downward. (**b**) The topographical maps of the difference ERPs. Displayed are the maps of real and pseudo- character conditions in the native (Lef t) and non-native (Right) group in the time windows of 120–160 ms, 170–210 ms and 340–380 ms.

Following the early window of 170–210 ms, the vMMN in real characters is sustained in a later time window of 340–380 ms. This effect is distributed in central-parietal areas, closely resembling that of the earlier window of 170–210 ms (See Fig. 1). This topographical similarity may imply overlapping lexical (-semantic) processing in the two intervals. Therefore, it is presumed that a secondary lexical reanalysis may occur in this interval. This effect is also consistent with previous auditory findings^4^, supporting the presence of biphasic MMNs in automatic lexical processing. In this interval of 340–380 ms, probably due to their established memory representations, real characters tend to maintain their advantage in vMMN elicitation against pseudo-characters, as shown by a marginally significant difference in the vMMN amplitudes of real and pseudo- characters. Still in this window, there was evidence of vMMN for pseudo-characters, in contrast to the effects in the earlier window of 170–210 ms. This could be due to availability of a certain degree of attentional resources in the later window, although still in a passive viewing context. If this is the case, there may thus be some activation of the lexical neighbours of the pseudo-characters^51^, leading to their vMMN. In fact, it has been shown in the auditory mode that MMN in the later windows (af ter 300 ms) is subject to attentional modulation such that with attentional resources, pseudoword MMN is increased to the extent of similar or larger amplitude to real word MMN, while real word MMN is found to be relatively immune to attentional variation^19,52^.

The dissociation of real and pseudo- characters in vMMN elicitation in our study is, however, in concordance with previous studies in the auditory modality which have consistently shown enlarged MMN effects elicited by real words in comparison with pseudowords, in different languages^4^. In terms of the time course of the effects, the present data show an early lexical effect of vMMN around 170 ms. This finding agrees well with previous lexical MMN investigations. For example, it was reported that the MMN amplitude produced by a syllable completing a real word was larger than that for a pseudoword at 150 ms af ter the stimulus uniqueness point^26^. Using a similar reverse oddball condition to our study, German words elicited enhanced MMN relative to matched pseudowords within the first 200 ms^36^. The authors argued that the pre-existing neuronal memory traces of real words are activated in an automatic manner and that the enhanced MMN component is a neurophysiological correlate of such activation. Through associative language learning experience over time, the neuronal network, represented as memory traces, becomes sufficiently robust to bolster rapid and automatic response to real words even in an attention-deprived condition^48^. Consistent with these auditory studies, our results lend support to this proposal and extend its applicability to the visual modality. The presence of vMMN for real characters also indicates that this component can be used as an index for higher-order language processing as well as for elementary visual feature processing^38^.

To the best of our knowledge, this is the first time that lexicality effects on vMMN elicitation have been observed for written language processing. These effects cannot be attributed to physical differences between the stimuli. As argued above, the real and pseudo-characters were matched on overall orthographic similarity, using the same set of semantic and phonetic radicals. In addition, they shared a context where five tokens of non-characters were used as fillers. Therefore, there were no differences in terms of contextual influence. The lexical vMMN effect in the current experiment was obtained with careful consideration to avoid potentially confounding factors. In this study, five tokens for each stimulus condition are accommodated, instead of the single token per stimulus type of ten used in previous auditory MMN studies. Multiple tokens can introduce more variability in basic visual features where different neuronal populations are stimulated and so have the advantage of reducing the stimulus-relevant refractoriness effect^53^. This practice can also prevent the local adaptation effects which contribute to deviance-related effects^38^. Furthermore, it is necessary to minimise any physical differences which may confound vMMN effects. In the current study, based on the orthographic features of Chinese characters, care was taken to build real and pseudo-characters, which were matched in overall visual-orthographic attributes. Specifically, exactly the same radical identities were used to build the two types of characters. Moreover, the radical position information, an important characteristic of Chinese characters, also remained unchanged. Only the pairings of semantic and phonetic radicals in real characters were changed to create pseudo-characters. This way, the lexical status (real vs. pseudo- character) was manipulated at a minimum price of physical change and confounding factors of physical feature difference can reasonably be eliminated in explaining the lexical vMMN effect. Finally, a non-linguistic distraction task in the foveal area was implemented to avoid attention to the vMMN-eliciting stimuli in the periphery.

In sum, the current study demonstrated how lexical information in Chinese is automatically and rapidly extracted, by exploring whether the previously-documented auditory linguistic MMN effect could be replicated in a visual modality. In native Chinese readers, single-character words and word-like stimuli both elicited vMMN effects compared with non-words in 120–160 ms and 340–380 ms, but only real words elicited vMMN in a later window of 170–210 ms. In the control group of non-readers of Chinese, there was no sign of vMMN for either real or pseudo- words. Our results extend previous findings on auditory lexical MMN effects and suggest that the underlying memory representations for real words may drive early lexical processing independently of presentation modality. While the present study does not investigate the neuroanatomical bases of the vMMN effects, future efforts may uncover their cortical underpinnings, using fMRI among other techniques.

## Methods

### Participants

Twenty-four native readers of Chinese and 19 non-Chinese-reading participants volunteered to take part in the experiment. All were neurologically healthy, with normal or corrected-to-normal vision and right-handed according to the Edinburgh Handedness Inventory^54^. The research protocol was approved by the Research Ethics Committees of the State Key Laboratory of Cognitive Neuroscience and Learning, Beijing Normal University, China and the University of Nottingham Ningbo, China. The methods carried out in the current study are in accordance with the approved guidelines and regulations. All participants provided informed written consent before taking part in the experiment and attended a brief post-experiment interview session, providing feedback about their experience of the experiment. Data from three Chinese participants were rejected due to their self-report of occasional active attention to the lexical stimuli, or due to an incomplete experiment. Another four participants were not included due to an excessive number of artifacts in the register. All the remaining 17 Chinese participants reported no awareness of the exact nature of the experiment and no notice of the regularity of oddball presentation of the to-be-ignored stimuli. The final sample of this native group, therefore, included 17 participants (aged 22.5 ± 2.2 years; 6 males). For the non-native group, data from two participants were rejected due to eye discomfort or active attention to linguistic stimuli. This resulted in a sample of 17 people (aged 26.5 ± 7.8, 7 males). All of these participants had lived in China for at least six months on an English-speaking campus and reported limited experience of the Chinese written language. Specifically, ten participants in the group had a total of 60–120 hours of classroom learning of Chinese in the past three years while the other seven had no formal training in the language but reported that they had picked up some Chinese characters incidentally (not the critical stimuli in the experiment) when interacting with their friends in China.

### Stimuli and procedure

Three types of stimuli, real, pseudo- and non- characters with five exemplars of each were prepared (See Table 2 for all the stimuli). Importantly, in order to reduce physical differences between them, the same semantic and phonetic radicals were used across the three types of stimuli. Real characters used in the experiment are existing Chinese characters, with an average frequency of 83.80 per million and an average length of 8.3 strokes, according to Chinese Single-character Word Database (www.personal.psu.edu/pul8/psylin-norm/psychnorms.html). Pseudo-characters were built by exchanging the semantic and phonetic radicals of real characters while maintaining their conventional radical positions. The Xinhua Zidian (*Xinhua Character Dictionary*)^55^ confirmed that the pseudo-characters are non-existent. Five non-characters were also invented by reversing the positions of semantic and phonetic radicals of real characters. These non-characters were used as fillers in the experiment.

**Table 2:** Three different types of stimuli.

Stimuli	1	2	3	4	5
Real character					
(Pinyin)	wu4	gang1	jie2	shen2	fan4
(Gloss)	*stuff*	*steel*	*knot*	*god*	*meal*
Pseudo-character					
Non-character					

As explained above, using the same radicals in the three types of characters enabled maximal similarity in terms of visual-orthographical analysis. Despite the physical feature similarity, the different pairings of radicals resulted in real, pseudo- and non-characters respectively. The lexical status difference was further validated by a lexical status norming test administered to the participants af ter the electroencephalogram (EEG) recording session. The task of participants was to judge to what extent the character presented was a meaningful real character on a seven-point Likert scale (7 = most likely to be a real character). A mixed design repeated measures ANOVA of Group (native vs. non-native) and Lexicality (Real vs. Pseudo- vs. non- characters) showed a main effect of Lexicality (F(2, 64) = 149.72, p < 0.001, partial η^2^ = 0.82) and an interaction of Lexicality and Group (F(2, 64) = 57.76, p < 0.001, partial η^2^ = 0.64). Further analysis of the two-way interaction was carried out to reveal any differences between the three lexical conditions in the two groups. For the native group, the three lexical conditions were significantly different from each other in rating (ps < 0.001, Mean rating score (real vs. pseudo- vs. non- characters): 7.00 vs. 4.02 vs. 1.06). For the non-native group, there was no difference revealed between real and pseudo- characters (p = 1.00, real vs. pseudo, 5.08 vs. 4.82), though both of them were rated more real-character-like than non-characters (real vs. non, 5.08 vs. 3.68, p < 0.001; pseudo vs. non, 4.82 vs. 3.68, p = 0.013). This result confirmed that the non-native participants have limited proficiency in Chinese character reading. Four oddball blocks were built and run randomly. They included: B1, standard real character vs. deviant non-character (RN); B2, standard non-character vs. deviant real character (NR); B3, standard non-character vs. deviant pseudo-character (NP); B4, standard pseudo-character vs. deviant non-character (PN). In each block, standards and deviants were presented with the restriction of no identical stimulus type or stimulus identity appearing sequentially and no fewer than two standards between any consecutive deviants. Each of the four blocks was initiated by three standards. Altogether in one block, 540 standards and 90 deviants were presented, evenly represented by five exemplars of each character type.

**Figure 2:**
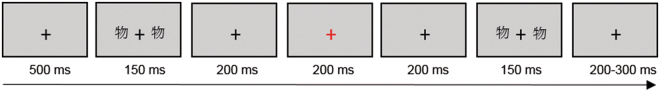
Illustration of the experimental procedure. Participants were asked to detect fixation colour change as accurately and quickly as possible and to memorise how many times they saw a red fixation cross in a block.

As shown in Fig. 2, each trial started with a fixation cross displayed for 500 ms in the center of a grey screen background, followed by two copies of a character (visual angles: 3.5 degrees horizontally and vertically; font: Sinsum; color: black) presented perifoveally both to the right and to the lef t of the cross. The distance from the centre of each character to the fixation centre was 7.2 degrees horizontally. The cross and characters remained on the screen for 150 ms, followed by an inter-stimulus interval of 600 ms where the black cross changed to red occasionally and unpredictably. This was followed by another presentation of a character, with the same configurations as the previous one. Then a blank screen with a random duration of 200 to 300 ms concluded one trial. In order to maintain participant attention to the screen centre and not to the stimuli in the peripheral area, fixation crosses, black or red, were always displayed in the middle of the screen. During the experiment, participants were seated in a comfortable chair at a viewing distance of 50 cm. They were instructed to focus their attention on detecting the red crosses in the centre of the screen, and press the “D” key on the keyboard as quickly and accurately as possible when they saw a red cross. Response time and accuracy were recorded by E-prime (Version 2.0, Psychology Sof tware Tools Inc., USA) and analyzed. In addition, they were asked to mentally count how many times they saw a red cross and report this at the end of the each four sessions in the experiment. Thus, a relatively stringent control of attention away from the lexical stimuli was maintained. Af ter the experiment, participants were asked to join in a post-experiment session which included a short interview as well as a short lexicality norming study (See above).

### Data recording and analysis

**Figure 3:**
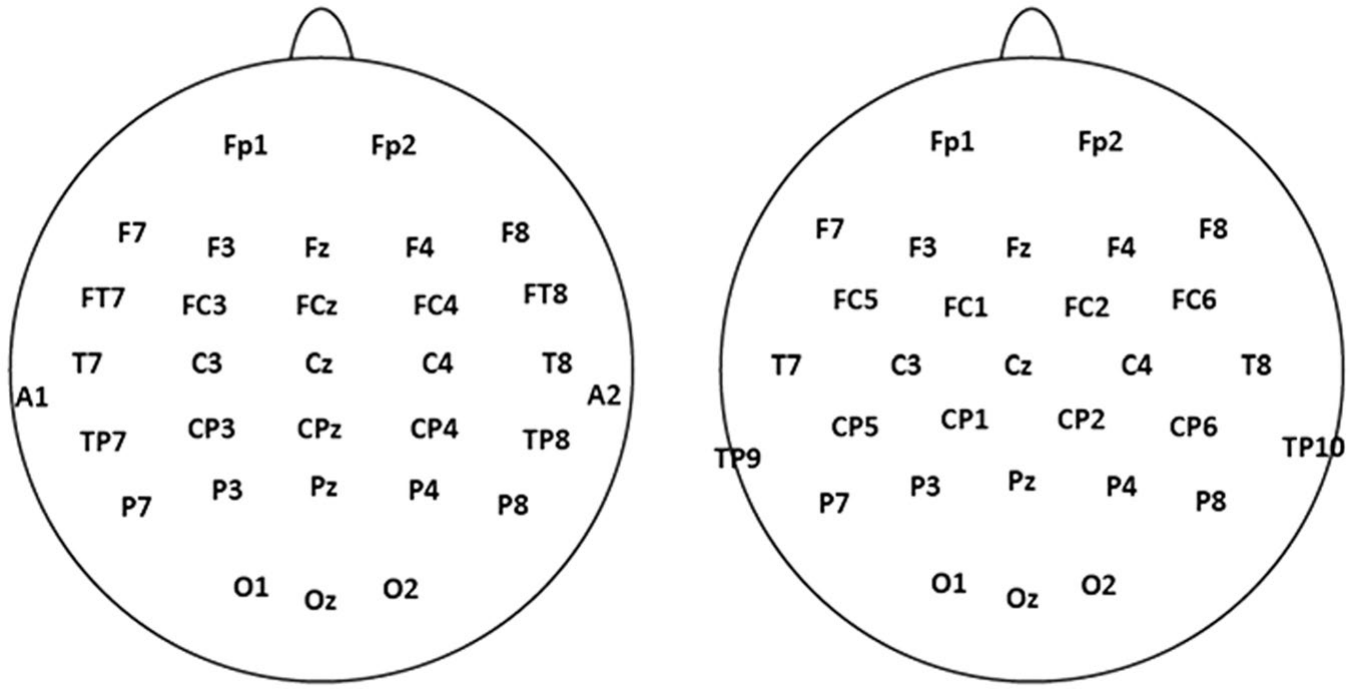
The two electrode caps used on the native and non-native group. Lef t: the cap used on the native group; Right: the cap used on the non-native group. Note that the channel layouts for the two groups are not identical.

Two EEG recording systems were used in the study. For the native group, EEG was registered using a Synamps amplifier (Compumedics NeuroScan, El Paso, TX) with a 32-channel electrode cap (QuickCap, Neuromedical Supplies, VA, USA) (Fig. 3 Lef t). For the non-native group, the BrainAmp DC amplifier plus a 32-channel EasyCap (Brain Products, Germany) (Fig. 3 Right) was used. Note that the layouts of the electrodes in the two caps are not identical. For both groups, the identical sampling rate of 500 Hz and band-pass of 0.05–100 Hz were adopted. All electrodes were referenced to the tip of the nose. The vertical and horizontal electrooculograms (EOGs) were monitored by electrodes placed at the outer canthus of each eye and those above and below the lef t eye respectively. All impedances were maintained at or below 5 kΩ. Offline, Brain Vision Analyzer (Version 2.1) was used for processing the data of the two groups. First, they were digitally bandpass-filtered between 0.1–30 Hz (24 dB/Octave) using a finite impulse response (FIR) filter (Butterworth zero phase filter). Second, a semiautomatic raw data inspection was carried out to delete bad intervals. To be specific, bad intervals were automatically marked if they met the following two criteria: 1) the maximum absolute difference of values surpassed 100 µV within an interval of not exceeding 50 ms; 2) the amplitude was not in the range of −100 to 100 µV in any channels except EOG channels. Then a careful manual check of the marked intervals and neighbouring deflections was followed to ensure all artifacts were cleaned up. Third, in order to reduce as much as possible the effect of eye movements on the EEG data, ocular correction was conducted according to the algorithm of Gratton, Coles and Donchin^56^. Fourth, the data were segmented from −150 to 600 ms relative to the onset of stimuli and corrected with a pre-stimulus baseline of 150 ms. Last, an additional step of artifact rejection in a semi-automatic mode was carried out to eliminate any artifact lef t undetected previously before the segmented data were averaged. In this step of artifact rejection, any potential artifact was marked based on the following criteria: 1), the differences in voltage between two data points were larger than 50 µV/ms; 2), the amplitude was not in the range of −80 to 80 µV at any channel except EOG channels. Then a manual inspection of the marked artifacts and neighbouring data was conducted to eliminate artifacts. The trials of deviants and standards of real and pseudo-characters were averaged for further analysis. In averaging, (1) the first 3 trials in each block, (2) trials where participants gave a response, and (3) the trials where there were cross color changes, were excluded. As a result, the mean number of accepted segments for final analysis was 73, 325, 74 and 325 for natives and 77, 392, 76 and 381 for non-natives, corresponding to deviant/standard real characters and deviant/standard pseudo-characters, respectively.

**Figure 4:**
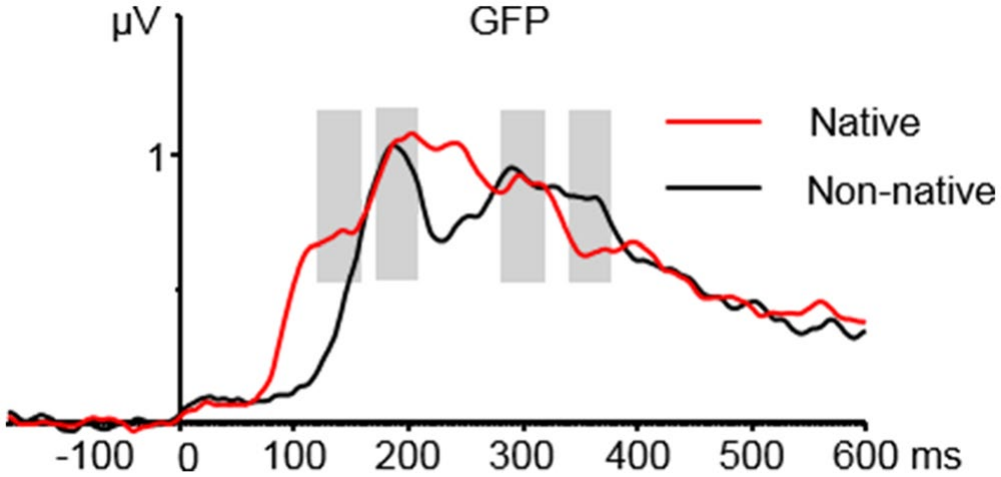
Overall activation strength shown in Global Field Power (GFP). Four intervals were selected covering the prominent peaks of the native group (Red) and the non-native group (Black): 120–160 ms, 170–210 ms, 280–320 ms and 340–380 ms.

In order to enable a bias-free choice of time windows for statistical analysis, we relied on (1) the overall activity strength of all electrodes (except vertical and horizontal EOGs) by computing the global field power (GFP)^57^ of the ERP responses of the two groups (Fig. 4) and (2) references on windows in previous language vMMN studies^27,35^. The GFP method, and a similar approach, using root mean square (RMS) were adopted in recent studies on language vMMN^27^ and early lexical processing^42,58^. Specifically, in the native group of our experiment, the ERPs to all four conditions of deviant real/pseudo- words and standard real/pseudo- words were collapsed and averaged for each electrode at each time point, producing a single response. Then the GFP of each participant was grand-averaged, which resulted in a final global GFP. The same steps were followed for the GFP of the non-native group. The prominent peaks in the two groups were identified and taken into account for the choice of windows. With this procedure and with reference to previous studies^27,35^, four windows were selected: 120–160 ms, 170–210 ms, 280–320 ms and 340–380 ms.

**Figure 5:**
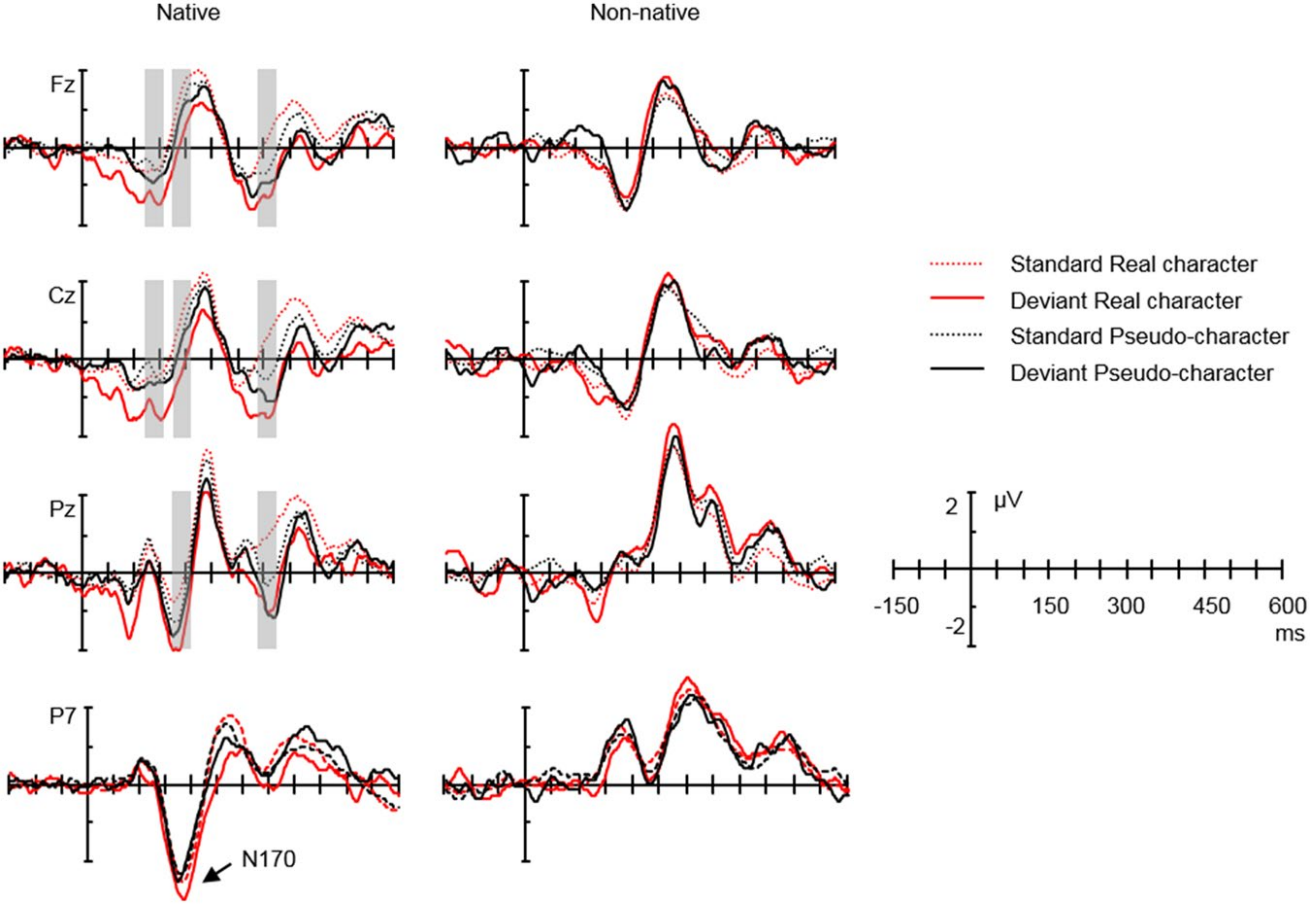
Comparison of deviant and standard stimuli. ERP waveforms for deviant (Red solid) and standard (Red dotted) real character condition and for deviant (Black solid) and standard (Black dotted) pseudo-character condition at the representative electrodes Fz, Cz, Pz for the native group (Lef t) and the non-native group (Right). Lef t corner: N170 on the electrode P7 for the native group. Negativity is plotted downward.

The effect of stimulus type on ERPs was analysed using a mixed-design ANOVA of Group (native vs. non-native), Type (deviant vs. standard), Lexicality (real vs. pseudo-character) and Region. With reference to previous language vMMN studies^27,34,35^, the topographical factor of Region was defined as frontal (F3/4, FC3/4, Fz, FCz), central (C3/4, CP3/4, Cz, CPz) and parietal (P3/4, O1/2, Pz, Oz) areas for the native group. The choice of electrodes in a region in the non-native group was adapted to that of the native group due to their slight difference in electrode layout (Fig. 3). The mean voltages of FC1/2 and CP1/2 were taken as an equivalent of FCz and CPz in the native group respectively. The Region in the non-native group was then realised as frontal (F3/4, FC5/6, Fz, FCz), central (C3/4, CP5/6, Cz, CPz) and parietal (P3/4, O1/2, Pz, Oz). If the above general ANOVA revealed evidence of vMMN (differences between deviants and standards) for both real and pseudo- characters, a following ANOVA of Lexicality (real vs. pseudo characters difference ERPs) and Region was carried out to explore any differences in vMMNs between the two lexical categories. See Fig. 5 for the deviant and standard ERPs of the two groups and Fig. 1 for the corresponding difference ERPs (Fig. 1a) and topographical maps (Fig. 1b).

In the native Chinese group, for the deviants and standards of both real and pseudo- characters, typical negative deflections peaking around 170 ms at parietal-occipital sites, resembling N170, were observed (Fig. 5 Lef t corner). In contrast, in a similar window, positive going waveforms were found for the non-native group at the posterior site (Fig. 5 Right corner). In order to explore the lateralisation of N170 and any differences between the two groups, amplitude measurements were submitted to an ANOVA of Group, Type (deviants vs. standards), Lexicality (real vs. pseudo- character) and Hemisphere (lef t: O1, P3, P7 vs. right: O2, P4, P8). In all the above analyses, where appropriate, critical values were adjusted using the Greenhouse-Geisser correction for violation of the assumption of sphericity. Hochberg-corrected p values were reported.

### Data Availability

The datasets generated during the current study are available from the corresponding author on reasonable request.

## References

[1] Brown C, Hagoort P (1993). The processing nature of the N400: Evidence from masked priming. J. Cogn. Neurosci..

[2] Kutas, M., Van Petten, C. & Kluender, R. In *Handbook of psycholinguistics* (eds Traxler, M. & Gernsbacher, M. A.) 659–724 (Elsevier, 2006).

[3] Hauk O, Pulvermüller F (2004). Neurophysiological distinction of action words in the fronto‐central cortex. Hum. Brain Mapp..

[4] Pulvermüller F, Shtyrov Y (2006). Language outside the focus of attention: The mismatch negativity as a tool for studying higher cognitive processes. Prog. Neurobiol..

[5] Barber HA, Kutas M (2007). Interplay between computational models and cognitive electrophysiology in visual word recognition. Brain Res. Rev..

[6] Pulvermüller F, Shtyrov Y, Hauk O (2009). Understanding in an instant: Neurophysiological evidence for mechanistic language circuits in the brain. Brain Lang..

[7] Shtyrov Y (2010). Automaticity and attentional control in spoken language processing: Neurophysiological evidence. Ment. Lexicon.

[8] Bentin S, Mouchetant-Rostaing Y, Giard MH, Echallier JF, Pernier J (1999). ERP manifestations of processing printed words at different psycholinguistic levels: time course and scalp distribution. J. Cogn. Neurosci..

[9] Pulvermüller, F. Word processing in the brain as revealed by neurophysiological imaging in *The* Oxford *Handbook of Psycholinguistics* (ed. Gaskell, M. G.) 119–139 (Oxford University Press, 2007).

[10] Coch D, Meade G (2016). N1 and P2 to words and wordlike stimuli in late elementary school children and adults. Psychophysiology..

[11] Coch D, Mitra P (2010). Word and pseudoword superiority effects reflected in the ERP waveform. Brain Res..

[12] Coch D, Mitra P, George E (2012). Behavioral and ERP evidence of word and pseudoword superiority effects in 7- and 11-year-olds. Brain Res..

[13] Compton PE, Grossenbacher P, Posner MI, Tucker DM (1991). A cognitive-anatomical approach to attention in lexical access. J. Cogn. Neurosci..

[14] McCandliss BD, Posner MI, Givon T (1997). Brain plasticity in learning visual words. Cogn. Psychol..

[15] Simon, G., Bernard, C., Largy, P., Lalonde, R. & Rebai, M. Chronometry of visual word recognition during passive and lexical decision tasks: an ERP investigation. *Int. J. Neurosci*. **114** (2004). 10.1080/00207450490476057 15636353

[16] McCandliss BD, Cohen L, Dehaene S (2003). The visual word form area: expertise for reading in the fusiform gyrus. Trends Cogn. Sci..

[17] Mahé G, Bonnefond A, Gavens N, Dufour A, Doignon-Camus N (2012). Impaired visual expertise for print in French adults with dyslexia as shown by N170 tuning. Neuropsychologia..

[18] Czigler I (2007). Visual mismatch negativity: violation of nonattended environmental regularities. J. Psychophysiol..

[19] Shtyrov Y, Kujala T, Pulvermüller F (2010). Interactions between language and attention systems: early automatic lexical processing?. J. Cogn. Neurosci..

[20] Näätänen R, Gaillard AW, Mäntysalo S (1978). Early selective-attention effect on evoked potential reinterpreted. Acta Psychol. (Amst.).

[21] Näätänen R (1990). The role of attention in auditory information processing as revealed by event-related potentials and other brain measures of cognitive function. Behav. Brain Sci..

[22] Paavilainen P (2013). The mismatch-negativity (MMN) component of the auditory event-related potential to violations of abstract regularities: A review. Int. J. Psychophysiol..

[23] Korpilahti P, Krause CM, Holopainen I, Lang AH (2001). Early and late mismatch negativity elicited by words and speech-like stimuli in children. Brain Lang..

[24] Pulvermüller F, Shtyrov Y, Kujala T, Näätänen R (2004). Word-specific cortical activity as revealed by the mismatch negativity. Psychophysiology..

[25] Sittiprapaporn W, Chindaduangratn C, Tervaniemi M, Khotchabhakdi N (2003). Preattentive Processing of Lexical Tone Perception by the Human Brain as Indexed by the Mismatch Negativity Paradigm. Ann. N. Y. Acad. Sci..

[26] Pulvermüller F (2001). Memory Traces for Words as Revealed by the Mismatch Negativity. Neuroimage..

[27] Shtyrov Y, Goryainova G, Tugin S, Ossadtchi A, Shestakova A (2013). Automatic processing of unattended lexical information in visual oddball presentation: neurophysiological evidence. Front. Hum. Neurosci..

[28] Czigler I, Balazs L, Winkler I (2002). Memory-based detection of task-irrelevant visual changes. Psychophysiology..

[29] Mo L, Xu G, Kay P, Tan LH (2011). Electrophysiological evidence for the left-lateralized effect of language on preattentive categorical perception of color. Proc. Natl. Acad. Sci..

[30] Kimura M, Katayama Ji, Ohira H, Schröger E (2009). Visual mismatch negativity: New evidence from the equiprobable paradigm. Psychophysiology..

[31] Kuldkepp, N., Kreegipuu, K., Raidvee, A., Näätänen, R. & Allik, J. Unattended and attended visual change detection of motion as indexed by event-related potentials and its behavioral correlates. *Front. Hum. Neurosci*. **7** (2013). 10.3389/fnhum.2013.00476 PMC3743214 23966932

[32] Stefanics G, Kremláček J, Czigler I (2014). Visual mismatch negativity: A predictive coding view. Front. Hum. Neurosci..

[33] Thierry G, Athanasopoulos P, Wiggett A, Dering B, Kuipers JR (2009). Unconscious effects of language-specific terminology on preattentive color perception. Proc. Natl. Acad. Sci..

[34] Wang X, Liu A, Wu Y, Wang P (2013). Rapid Extraction of Lexical Tone Phonology in Chinese Characters: A Visual Mismatch Negativity Study. PLoS One.

[35] Wang X, Wu Y, Liu A, Wang P (2013). Spatio-temporal dynamics of automatic processing of phonological information in visual words. Sci. Rep..

[36] Endrass T, Mohr B, Pulvermüller F (2004). Enhanced mismatch negativity brain response after binaural word presentation. Eur. J. Neurosci..

[37] Kecskés-Kovács K, Sulykos I, Czigler I (2013). Visual mismatch negativity is sensitive to symmetry as a perceptual category. Eur. J. Neurosci..

[38] Stefanics G, Csukly G, Komlósi S, Czobor P, Czigler I (2012). Processing of unattended facial emotions: A visual mismatch negativity study. Neuroimage..

[39] Taft M, Zhu X (1997). Submorphemic processing in reading Chinese. J. Exp. Psychol. Learn. Mem. Cogn..

[40] Feldman LB, Siok WWT (1999). Semantic Radicals Contribute to the Visual Identification of Chinese Characters. J. Mem. Lang..

[41] Lin SE (2011). Left-lateralized N170 response to unpronounceable pseudo but not false Chinese characters—the key role of orthography. Neuroscience..

[42] Maurer U, Zevin JD, McCandliss BD (2008). Left-lateralized N170 effects of visual expertise in reading: evidence from Japanese syllabic and logographic scripts. J. Cogn. Neurosci..

[43] Maurer U, Brandeis D, McCandliss BD (2005). Fast, visual specialization for reading in English revealed by the topography of the N170 ERP response. Behav. Brain. Funct..

[44] Proverbio AM, Leoni G, Zani A (2004). Language switching mechanisms in simultaneous interpreters: an ERP study. Neuropsychologia..

[45] Yum YN, Su I-F, Law S-P (2015). Early Effects of Radical Position Legality in Chinese: An ERP Study. Sci. Stud. Read..

[46] Hauk O (2006). [Q:] When would you prefer a SOSSAGE to a SAUSAGE?[A:] At about 100 msec. ERP correlates of orthographic typicality and lexicality in written word recognition. J. Cogn. Neurosci..

[47] Ding G, Peng D, Taft M (2004). The nature of the mental representation of radicals in Chinese: a priming study. J. Exp. Psychol. Learn. Mem. Cogn..

[48] Shtyrov Y, Pulvermüller F (2007). Language in the mismatch negativity design: motivations, benefits, and prospects. J. Psychophysiol..

[49] Marslen-Wilson WD (1987). Functional parallelism in spoken word-recognition. Cognition.

[50] Shu, H. & Anderson, R. C. In *Reading* Chinese *script: A cognitive analysis* (eds Wang, J., Chen, H. C., Radach, R. & Inhoff, A.) 1–18 (Lawrence Erlbaum, 1999).

[51] Yap MJ, Lim GY, Pexman PM (2015). Semantic richness effects in lexical decision: The role of feedback. Mem. Cognit..

[52] Garagnani, M., Shtyrov, Y. & Pulvermüller, F. Effects of attention on what is known and what is not: MEG evidence for functionally discrete memory circuits. *Front. Hum. Neurosci*. **3** (2009). 10.3389/neuro.09.010.2009 PMC2715270 19680433

[53] May PJ, Tiitinen H (2010). Mismatch negativity (MMN), the deviance-elicited auditory deflection, explained. Psychophysiology..

[54] Oldfield, R. C. The assessment and analysis of handedness: the Edinburgh inventory. *Neuropsychologia*. **9** (1971). 10.1016/0028-3932(71)90067-4 5146491

[55] Editorial-Board. *New China Character Dictionary*. 10th edn, (The Commercial Press, 2005).

[56] Gratton G, Coles MG, Donchin E (1983). A new method for off-line removal of ocular artifact. Electroencephalogr. Clin. Neurophysiol..

[57] Lehmann D, Skrandies W (1980). Reference-free identification of components of checkerboard-evoked multichannel potential fields. Electroencephalogr. Clin. Neurophysiol..

[58] Wang F, Maurer U (2017). Top-down modulation of early print-tuned neural activity in reading. Neuropsychologia..

